# Spectrum and Sensitivity of Bacterial Keratitis Isolates in Auckland

**DOI:** 10.1155/2016/3769341

**Published:** 2016-04-26

**Authors:** S. Marasini, S. Swift, S. J. Dean, S. E. Ormonde, J. P. Craig

**Affiliations:** ^1^Department of Ophthalmology, New Zealand National Eye Centre, The University of Auckland, Private Bag Box 92019, Auckland 1142, New Zealand; ^2^Department of Molecular Medicine and Pathology, The University of Auckland, Private Bag Box 92019, Auckland 1142, New Zealand

## Abstract

*Background.* The bacteria isolated from severe cases of keratitis and their antibiotic sensitivity are recognised to vary geographically and over time.* Objectives.* To identify the most commonly isolated bacteria in keratitis cases admitted over a 24-month period to a public hospital in Auckland, New Zealand, and to investigate* in vitro* sensitivity to antibiotics.* Methods.* Hospital admissions for culture-proven bacterial keratitis between January 2013 and December 2014 were identified. Laboratory records of 89 culture positive cases were retrospectively reviewed and antibiotic sensitivity patterns compared with previous studies from other NZ centres.* Results.* From 126 positive cultures, 35 species were identified.* Staphylococcus* was identified to be the most common isolate (38.2%), followed by* Pseudomonas* (21.3%). Over the last decade, infection due to* Pseudomonas* species, in the same setting, has increased (*p* ≤ 0.05). Aminoglycosides, cefazolin, ceftazidime, erythromycin, tetracycline, and doxycycline were 100% effective against tested isolates* in vitro*. Amoxicillin (41.6%), cefuroxime (33.3%), and chloramphenicol (94.7%) showed reduced efficacy against Gram-negative bacteria, whereas penicillin (51%) and ciprofloxacin (98.8%) showed reduced efficacy against Gram-positive bacteria.* Conclusions.* Despite a shift in the spectrum of bacterial keratitis isolates, antibiotic sensitivity patterns have generally remained stable and show comparability to results within the last decade from NZ centres.

## 1. Introduction

Microbial keratitis (MK) is an ocular emergency that requires prompt and specific management to maximise visual outcome and preserve ocular integrity. Although there is variability in the reported rates [1], it appears that the rate of this potentially devastating disease has risen in the last 50–60 years [2, 3]. This increase in incidence has been attributed to specific risk factors such as the use of contact lens [3, 4]. Bacterial keratitis (BK) is the most common form of MK. To date, 77 species from 42 genera of bacteria have been identified to have a potential to cause keratitis [5]. This diversity of organisms contributes to the need for targeted treatment. The treatment regimen of BK has long been debated with one school of thought endorsing corneal scraping and combination antibiotic therapy and the other empirical fluoroquinolone therapy alone [6, 7]. Guidelines have been published for the treatment of the disease, with equal priority assigned to the laboratory results and the clinical impression, severity of the condition, and the pathogen [8, 9]. The treatment protocol based on clinical diagnosis alone has the benefit of allowing rapid intervention but does not provide confirmation of the causative organism which can otherwise be hard to identify due to overlapping clinical signs from different pathogens [6]. Furthermore, confounding factors such as inappropriate treatment (e.g., corticosteroids) can lead to severe complications [10].

Treatment based on laboratory culture has the advantage that a positive culture together with antibiotic sensitivities allows the selection of the appropriate class of antibiotics, thus avoiding unnecessary use of ineffective and potentially toxic drugs [6, 11]. Moreover if a corneal scrape is performed, it can facilitate healing through superior antibiotic penetration and debridement of necrotic tissues [9]. The downside, however, is that there is a delay in culturing bacteria and refining sensitivities, and therefore, commonly a dual approach is taken whereby empirical therapy is commenced following corneal scraping and treatment is refined as necessary according to clinical response and laboratory-based sensitivity data.

In the present study, the ocular microbiological data for all MK cases admitted to Greenlane Clinical Centre (GCC), Auckland, New Zealand (NZ), over the 2-year period, 2013 to 2014, were reviewed, to determine the bacterial species causing keratitis and their susceptibility to antibiotics in laboratory testing. Findings were further compared to those reported in the literature in 2003, which relate to the same catchment area over the period 1999–2001, to help identify local aetiological trends, confirm sensitivity patterns to commonly prescribed antibiotics, and inform protocols for optimal management of the disease.

## 2. Methods

An institutional review board-approved (UAHPEC 013549), retrospective review of culture-proven BK was undertaken for the period 1 January 2013 to 30 December 2014 at GCC. Patients hospitalized for intensive antibiotic treatment were identified through a computerized diagnostic code search and ward discharge record search (with keratitis listed as a discharge diagnosis). A single author (S. Marasini) reviewed the medical records to extract relevant information concerning the growth and susceptibility of pathogens. The microbiological laboratory records were reviewed for Gram stain, culture isolates, and antibiotic sensitivity results. While Gram stain results were usually available the same day, various media were routinely incubated for a total of 10 days to 2 weeks depending on the media before giving a final report. Possible bacterial contaminants (as indicated in the laboratory report) were excluded from analysis. All microbiological testing was carried out independently by the microbiology laboratory at GCC. Depending on the current laboratory protocols and the isolated organism, the following antimicrobial agents were included in susceptibility testing: penicillin (amoxicillin, amoxi/clavulanate, ticarcillin/clavulanate, penicillin, and flucloxacillin), fluoroquinolone (ciprofloxacin), cephalosporins (cefazolin, cefuroxime, and ceftazidime), aminoglycoside (tobramycin, gentamicin, and neomycin), chloramphenicol, and tetracycline.* In vitro* susceptibility was determined by the Kirby-Bauer disk diffusion method and Minimum Inhibitory Concentration (MIC). Isolates of intermediate sensitivity were categorized as susceptible organisms because the concentration of drug achieved at the ocular surface is much greater than that used for* in vitro* susceptibility testing [12]. Statistical analysis was performed using GraphPad Prism 6 software, using the Fisher exact test and statistical significance was set at *p* ≤ 0.05.

## 3. Results

There were 196 cases (194 patients) of severe microbial keratitis between January 2013 and December 2014. In 154 cases, a corneal scrape was performed, of which 89 (57.8%) grew organisms. Bacteria were isolated from all of these culture positive cases. Conjunctival swab culture was available in 11 cases and both corneal scrape and swab cultures were available for 126 cases. The remainder of the cases received treatment or were continued on treatment, without culture.

At least one risk factor was identified in 80.6% cases and included contact lens wear (33.1%), ocular surface disease of varying etiology (29.6%), previous ocular surgery (18.6%), trauma (14.8%), and steroid use (8.2%). Multiple risk factors were identified in 13.8% of cases and a cause could not be identified in 19.4%. Twenty-six patients were diabetic with (*n* = 16) or without (*n* = 10) known ocular risk factors.

Among the culture positive cases in the contact lens group (*n* = 25), 72% grew Gram-negative organisms, which included* Pseudomonas aeruginosa* (*n* = 14) and* Serratia* species (*n* = 5).* Staphylococcus* species were isolated in 5 cases. More than two organisms grew in 10 instances of contact lens wearers; three organisms grew in two and five organisms in one instance. In the cases in which* Moraxella* species grew (*n* = 16), risk factors identified were ocular surface disease (*n* = 5), prior ocular surgery (*n* = 3), and ocular trauma (*n* = 2).

Fungi and yeast were isolated in eight scrapes and* Acanthamoeba* was isolated in one. The species of fungi and yeast included* Fusarium solani* (2),* Candida albicans* (1),* Aspergillus fumigatus* (1),* Candida guilliermondii* (1),* Candida parapsilosis* complex (2), and* Epicoccum *species (1). In addition, herpes simplex virus (HSV) was isolated in 16 cases and herpes zoster virus (HZV) in two. Serology was positive for HSV/HZV in one instance.

A repeat scrape was performed in 16 cases where the first scrape was inconclusive. On rescrape, no growth was identified in seven but the remainder grew organisms such as* Corynebacterium* species (2),* Staphylococcus* species (2),* Streptococcus* species (3), and* Moraxella* species (2). Of all the* scrapes*, 89 (57.8%) yielded bacterial cultures growing 126 organisms: 73 (58%) Gram-positive and 53 (42%) Gram-negative. The range of bacteria isolated from the corneal scrapes is shown in Table 1. Gram stain from the scrapes was positive in 69 (44.8%). Twenty-nine cases grew more than one organism: two (*n* = 20), three (*n* = 5), four (*n* = 3), and more than four organisms (*n* = 1).

Conjunctival swab staining was performed in 137 samples with Gram stain positive in 55 (40.1%): 28 (50.9%) Gram-positive and 27 (49.1%) Gram-negative. Swab culture identified 23 organisms. In one case, swab culture and corneal scrape culture grew different organisms. In a number of cases, contact lenses were cultured (*n* = 16), contact lens cases were cultured (*n* = 7), or both were cultured (*n* = 5). Contact lens culture grew Gram-negative organisms in eight cases (*Pseudomonas aeruginosa* in five of these cases) and contact lens case culture grew* Pseudomonas aeruginosa* in one case. One contact lens culture did not grow any organism, and one grew an organism absent on corneal scrape. Thirty-five different bacterial species from 18 genera were isolated over the two-year period.* Staphylococcus* species were the most commonly isolated organisms (38.2%), followed by* Pseudomonas* species (21.3%),* Streptococcus* species (20.2%),* Moraxella* species (18%), and* Corynebacterium* species (16.9%). Together, these five groups accounted for 82.5% of the isolates.

Table 2 shows the* in vitro* susceptibility of Gram-positive and Gram-negative bacteria to a range of antibiotics tested during the study period. For Gram-negative bacteria, 100% of isolates were susceptible to tested aminoglycosides (gentamicin, neomycin, and tobramycin). Among the cephalosporins, 67% of Gram-negative isolates were resistant to cefuroxime but 100% were susceptible to cefazolin and ceftazidime. Ciprofloxacin had good coverage for Gram-negative pathogens (100%). All* Pseudomonas aeruginosa* isolates were sensitive to gentamicin, ceftazidime, and ciprofloxacin. All Gram-positive bacteria were susceptible to aminoglycosides, cephalosporins, vancomycin, chloramphenicol, erythromycin, flucloxacillin, and tetracycline. Some of the Gram-positive isolates exhibited resistance to ciprofloxacin (1.2%) and penicillin (49%). Antibiotic coverage against the most commonly isolated bacteria is shown in Table 3.

Based on the susceptibility test, the relative effectiveness of commonly used empirical antibiotics, including ciprofloxacin and a combination regimen, cefazolin with gentamicin or tobramycin for Gram-negative bacteria (*Pseudomonas aeruginosa* and non-*Pseudomonas*) was analysed. All the isolates were equally susceptible (100%) to ciprofloxacin and combination regimen of cefazolin and gentamicin (or tobramycin).

## 4. Discussion

The aim of the present study was to identify the current spectrum of bacteria causing keratitis leading to hospitalization in a tertiary care centre with a wide catchment area in Auckland, New Zealand, and to evaluate recent antibiotic susceptibility patterns. A wide range of pathogens was identified which showed variable susceptibility against tested antibiotics. Sixty-seven percent of isolates were resistant to cefuroxime, which forms the most common empirical dual therapy prescription along with tobramycin in our centre (unpublished data). Aminoglycosides (gentamicin, neomycin, and tobramycin) demonstrated high* in vitro* efficacy along with cefazolin, erythromycin, vancomycin, tetracycline, and doxycycline each at 100% compared to ciprofloxacin and chloramphenicol at rates of 98.8% and 94.7%, respectively.

BK requires treatment as an ocular emergency with the application of an effective antibiotic delivered at a sufficient dose, especially when large ulcers involve the visual axis [13]. The initial recommended treatment regimen in BK is either a fortified preparation of a cephalosporin (5%) with a fortified aminoglycoside (1.5% tobramycin or gentamicin) or fluoroquinolone monotherapy, until the corneal surface has been sterilized [6, 9]. However, this therapy is toxic in prolonged use, and sterility does not equate directly with healing or resolution of inflammation, reflected by healing and closure of the corneal epithelium [6]. In the last decade, initial empirical treatment in Auckland Hospital (NZ) has shifted from the first-generation fortified cephalosporin (cefazolin) together with the fortified aminoglycoside (gentamicin or tobramycin) towards a second-generation cephalosporin (cefuroxime) combined with an aminoglycoside (tobramycin). Cefazolin and ceftazidime were very effective (100%) against tested Gram-positive and Gram-negative bacteria. Indeed, for ceftazidime there has been no report of an increase in resistance since 1994 [14], and it was highly effective (100%) against* Pseudomonas aeruginosa* in the current study. Although dual therapy is commonly prescribed (64%) in Auckland [10], the* in vitro* sensitivity of cefuroxime was tested only in nine cases in which two-thirds showed resistance, differing from the findings (5% resistance) in Waikato (NZ) [15]. Very high* in vitro* resistance to cefuroxime (49.1%) has been recently reported (2011) from Oxford, UK [12], for the BK isolates for the decade 1999–2009, particularly within Gram-negative organisms (91.2%) (Table 4). Aminoglycosides such as gentamicin are commonly used to increase the antimicrobial efficacy in dual therapy, especially against species ofstreptococci. In the present study, BK isolates were highly susceptible to gentamicin, a finding consistent with reports from elsewhere [12]. We observed that the* in vitro* efficacy of dual therapy consisting of cefazolin and gentamicin (or tobramycin) was equally effective (100%) to ciprofloxacin monotherapy against* Pseudomonas aeruginosa* and other non-*Pseudomonas* Gram-negative bacteria. Neomycin exhibited 45.4% intermediate resistance to* Pseudomonas* isolates, however, based on our methodology; we have classified them as sensitive organisms.

The type and frequency of organisms causing BK in this study vary from other centres. Infections due to fungi and yeasts (8 cases) and* Acanthamoeba* (a single case) were uncommon, consistent with previous literature [10]. We found a higher rate of S*treptococcus* species compared to that reported from the same catchment area in Auckland in 2003 [10] and from other New Zealand catchments such as Wellington [17] and Waikato [15]. These rates are higher than those from Australia [18] and Taiwan [14]. The rate of isolation of* Staphylococcus* species was less than other centres but that of* Moraxella* and* Corynebacterium* species was comparable within centres in NZ (Table 5). Interestingly, polymicrobial infection is common in Auckland (32.5%), similar to the previous report (33.3%) [10] but approximately four times higher than that in Waikato (8.3%) [15]. The climate and latitude variations perhaps play a role in the type and frequency difference in organisms' isolation and the regional variations have been well documented previously [19]. We observed a higher isolation rate of* Pseudomonas* during the study period than other NZ cities [15, 16] and than the previous report from the same hospital (*p* = 0.05) [10] as well as in Australia (8.3%) [18], but much lower than that in Taiwan (46.7%) [14]. The rates of* Pseudomonas* isolation are observed to differ between locations such as in Los Angeles (57% and 43% at two locations) [13], Germany (10%), Brisbane (17%) [4], and China (20%) [20]. Factors found to be influencing* Pseudomonas* infections have been explored in several studies and one of the commonest risk factors is determined to be contact lens use [21]. In our study sample and consistent with previous studies [21], contact lens use was the most common risk factor (33.1%) followed by ocular surface disease of varying etiology (29.6%). In contact lens wearers, infections due to Gram-negative bacteria prevailed (72%), of which 78% arose from* Pseudomonas aeruginosa* alone.

Another notable observation was the reduced incidence of methicillin-resistant* Staphylococcus aureus* (MRSA) (one isolate), an organism which is most commonly isolated following ocular surgery, being more common in US provinces [23] and resistant to fourth-generation fluoroquinolone [24]. Interestingly, over the last decade, there was a significant decrease in the infections caused by coagulase-negative S*taphylococcus aureus* (Table 1).

In the present study, a positive culture rate on presentation was found to be 57.8%, which is less compared to that from other NZ cities that range from 58% to 85% [15–17], but similar to that from the UK (53.7%) [12]. Prior use of antibiotics or failure to cease antibiotic use prior to corneal scraping may result in a negative culture. Auckland Hospital is a tertiary referral centre; therefore, the prior use of topical antimicrobial therapy, especially in contact lens wearers, likely contributed to the observed variance at this centre. In fact, corneal scrapes from 40 contact lens wearers failed to grow any organism in this study. Moreover, topical anaesthetic agents applied before corneal scraping may also produce false negative results [25]. In 16 cases, the contact lens was cultured, including one case, which grew an organism that did not grow on the corneal scrape. This might suggest that invasive corneal rescraping might not be necessary if contact lenses/lens cases are cultured more frequently at presentation.

In the current study, conjunctival swab staining was positive in 40% of the samples tested and 23 (of 126) organisms grew in swab culture. The Gram stain of a conjunctival swab is not as sensitive as that of a corneal scrape or culture but does give an early indication regarding first-line treatment [8]. However, many community ophthalmologists overlook the potential benefit of these quick, less invasive, and relatively inexpensive techniques and begin an empiric approach to therapy, with the assumption that, regardless of the identity of the organism, broad spectrum antibiotic treatment has a high probability of success [26]. However, as a downside, this empiric practice has been suggested to be a factor contributing to antibiotic resistance [27].

The emergence of resistance to antimicrobial agents is a global public health concern and resistance patterns vary greatly with geographical location. Periodic susceptibility testing ensures that the antimicrobials being used still have good coverage against isolates of BK [14]. This data has not been reported for over a decade in Auckland. Our study found excellent susceptibilities to aminoglycosides (100%). These findings are similar to other studies, which report susceptibilities that range from 72% to 100% [12, 14, 15, 28, 29]. However, in Brazil, gentamicin and tobramycin have demonstrated decreased efficacy, from 95% to less than 80%, over a period of 15 years since 1985 [30]. A similar trend has also been reported in Taiwan [14]. Since the same class of antibiotics may exhibit different efficacies against BK isolates from different regions, the choice of an antibiotic should be based on a prudent analysis of the location as well as risk factors and the historical data.

Universally, fluoroquinolones are widely used as monotherapy, reportedly constituting 95% of all prescriptions for BK (80% of which were ciprofloxacin) in Australia during 2001–2003 (not limited to BK requiring hospital admission) [18]. Because of their broad spectrum that ranges from 72% to 99% [13–15, 28–30] and interest in emerging resistance patterns (mainly against Gram-positive cocci, ranging from 6% to 35%) [20, 31–33], fluoroquinolones have recently become one of the most intensely studied antibiotics. They exhibit equivalent efficacy when compared with the standard dual fortified antibiotic regime in terms of corneal healing and comfort factors, offering an effective alternative, particularly when compliance is the main concern. However, 30.7% of corneal isolates in India have been reported to show resistance to ciprofloxacin [31], with a similar picture in South Florida (11% in 1990 to 28% in 1998) [32] and Pittsburgh (from 5.8% in 1993 to 35.0% in 1997) [33]. In the present study, ciprofloxacin demonstrated 100% efficacy against Gram-negative bacteria, an observation similar to that from Waikato (99%) [15] and Christchurch (100%) in New Zealand [16]. However, one-Gram-positive pathogen (*Nocardia veteran*) showed resistance against ciprofloxacin. In Christchurch, 12% of Gram-positive bacteria were resistant to ciprofloxacin [16], which differs from the present study (1.2%). The observed trend of BK isolates developing resistance against ciprofloxacin in different study centres suggests that an alternative to ciprofloxacin should perhaps be considered. As ofloxacin has recognised associations with ocular perforation, alternative newer generation fluoroquinolones with demonstrated activity* in vitro* and* in vivo* against Gram-positive cocci need to be considered [34]. Kowalski et al. (2003) showed a greater* in vitro* efficacy of fourth-generation fluoroquinolones to* Staphylococcus aureus* resistant to ciprofloxacin, ofloxacin, and levofloxacin [34]. However, in the present study only ciprofloxacin was tested against the BK isolates. Zhang et al. (2008) reported resistance to the newer generation fluoroquinolone and levofloxacin, at a rate of 15.5% [20]. The increasing resistance of antibiotics (mainly fluoroquinolones) against BK isolates, especially in Indian subcontinents, is attributable to the widespread use of antibiotics for systemic infections and prophylaxis and the availablility of over-the-counter antibiotics [27]. In New Zealand, a survey (2003) was conducted to identify the prescribing trends in infectious keratitis and it was found that fluoroquinolone monotherapy was widely used to treat milder cases of keratitis [35]. Our results indicate that ciprofloxacin continues to be effective* in vitro* against 98.8% BK isolates and this has changed minimally over the last decade, according to reports from different cities in the country [15, 16]. Mather et al. (2002) showed that the fourth-generation fluoroquinolones were more potent against Gram-positive bacteria and were equally effective against Gram-negative bacteria compared to second- and third-generation fluoroquinolones [36]. Harmonizing this evidence [36] with other observations [34], fourth-generation fluoroquinolone (gatifloxacin and moxifloxacin) can perhaps be considered as an alternative to ciprofloxacin to treat BK at the present time, where resistance issues exist.

Among other antibiotics tested, vancomycin was found to be 100% effective against Gram-positive bacteria. In the literature, no resistance has been reported for vancomycin [14]. Chloramphenicol was deemed 100% effective against tested Gram-positive bacteria and showed 94.7% efficacy against Gram-negative bacteria, consistent with other NZ studies [15, 17]. In the UK, over the last decade, 32% BK isolates developed resistance against chloramphenicol, which is high compared to the reports from NZ cities, where the reported resistance is between 3.2% and 6.9% (Table 4) [12, 15–17]. Our study indicated low* in vitro* efficacy of penicillin against Gram-positive isolates.

The conclusions of this study should be considered in the context of methodological limitations. It must be noted that many organisms could not be included in the study because of failure to grow in the microbiology laboratory, possibly introducing some selection bias and possibly confounding the* in vitro* susceptibility results to a degree. This is no different to previous studies, however. Newer generation fluoroquinolones are found to be effective against BK isolates, but only ciprofloxacin was tested* in vitro*. Although it is found to be highly effective, a guarded use is advised in a context of global variation in efficacy against BK isolates. Further, this study is limited to the severe cases requiring hospital admission. Cases managed on an outpatient basis are therefore not included. Also the possibility that Gram-positive rods such as* Propionibacterium* species and Gram-positive cocci such as coagulase-negative* Staphylococcus *species may represent nonpathogenic commensal species from the ocular surface cannot be excluded.

Notwithstanding with these limitations, several conclusions can be drawn from this study. Firstly, the relative proportion of* Pseudomonas* infections is higher in Auckland compared to other NZ cities and compared to prior records from the same city. Secondly, the antibiotic sensitivity pattern has not changed significantly in New Zealand over the last decade. The* in vitro* efficacy of aminoglycosides, cefazolin, ceftazidime, vancomycin, and tetracycline remains high but BK isolates are observed to be developing resistance against cefuroxime. Finally, ciprofloxacin has demonstrated equivalent efficacy against BK isolates* in vitro* compared with dual therapy consisting of cefazolin and gentamicin (or tobramycin) combination.

## Figures and Tables

**Table 1:** Prevalence of bacteria isolated in corneal scrape from severe cases of keratitis in Auckland Hospital in New Zealand (2013-2014) compared with results at the same site in 1999–2001.

Organism	Positive cultures (% of total)^#^ (2013-2014)	Positive cultures (% of total)^#^ (1999–2001) [10]	Statistical comparison between two time periods (*p* value)
Gram-positive cocci	53 (59.5)	N/A	
Coagulase-negative *Staphylococcus *	2 (2.3)	20 (26.7)	0.00001
*Staphylococcus aureus*	14 (15.7)	7 (9.3)	0.17
*Staphylococcus epidermidis*	8 (8.9)	14 (18.7)	0.10
*Streptococcus pneumoniae*	10 (11.2)	11 (14.7)	0.64
Others^a^	19 (21.3)	N/A	
Gram-positive bacilli	19 (21.3)	N/A	
*Propionibacterium *species	3 (3.4)	17 (22.7)	0.0001
*Corynebacterium *species^b^	15 (16.9)	6 (8)	0.10
Others^c^	1 (1.1)	N/A	
Gram-negative bacilli	53 (59.5)	N/A	
*Pseudomonas aeruginosa*	19 (21.3)	7 (9.3)	0.05
*Moraxella *species^d^	16 (18)	6 (8)	0.06
*Serratia marcescens *	5 (5.6)	3 (4)	0.72
Others^e^	12 (13.5)	N/A	
Other bacteria	2 (2.2)	N/A	
*Microbacterium *species	1 (1.1)	N/A	
*Aggregatibacter aphrophilus*	1 (1.1)	N/A	
Polymicrobial infection	29 (32.5)	25 (33.3)	1.00
Positive culture	89/154 scrapes	75/105 scrapes	—

^a^ *Streptococcus mitis *(4)*, Staphylococcus capitis *(3)*, Staphylococcus warneri *(3)*, Streptococcus *Lancefield (3)*, Staphylococcus haemolyticus *(1)*, Parvimonas micra *(1)*, Streptococcus pyogenes *(1), methicillin-resistant* Staphylococcus aureus *(1)*, flucloxacillin-resistant Staphylococcus aureus *(1), and* Staphylococcus lugdunensis *(1).

^b^ *Corynebacterium macginleyi *(9),* Corynebacterium pseudodiphtheriticum *(2),* Corynebacterium jeikeium *(1), and other* Corynebacterium *species (3).

^c^ *Nocardia veterana *(1).

^d^ *Moraxella lacunata *(8),* Moraxella nonliquefaciens *(6), and other* Moraxella *species (2).

^e^ *Haemophilus influenza *(4)*, Klebsiella oxytoca *(2),* Shewanella putrefaciens *(1),* Elizabethkingia meningoseptica *(1)*, Acinetobacter baumannii* complex (1), *Stenotrophomonas maltophilia *(1)*, Proteus mirabilis *(1), and* Morganella morganii *(1).

^#^Percentage sum was greater than 100% because of polymicrobial infections; N/A = not available.

**Table 2:** Antibiotic susceptibility of bacteria identified in microbial keratitis in Auckland Hospital in New Zealand (2013-2014)^a^.

Antibiotics	Gram-negative bacteria *N* (%)	Gram-positive bacteria *N* (%)	Total sensitivity (%)
Aminoglycosides			
Gentamicin	29 (100)	2 (100)	100
Neomycin	27 (100)	26 (100)	100
Tobramycin	27 (100)	27 (100)	100
Cephalosporins			
Cefazolin	12 (100)	16 (100)	100
Cefuroxime^b^	3 (33.3)	0 (0)	33.3
Ceftazidime	22 (100)	2 (100)	100
Fluoroquinolones			
Ciprofloxacin^c^	41 (100)	38 (98.8)	98.8
Penicillin group			
Penicillin^d^	1 (7.7)	29 (50.9)	42.9
Amoxicillin^e^	5 (41.6)	0 (0)	41.6
Flucloxacillin	4 (100)	29 (100)	100
Others			
Chloramphenicol^f^	18 (94.7)	23 (100)	94.7
Erythromycin	5 (100)	51 (100)	100
Tetracycline	2 (100)	14 (100)	100
Doxycycline	4 (100)	43 (100)	100
Vancomycin	0 (0)	29 (100)	100

^a^For each bacterial isolate, only selected antibiotics were tested.

^b^6 Gram-negative organisms were resistant.

^c^1 Gram-positive organism was resistant.

^d^12 Gram-negative and 28 Gram-positive organisms were resistant.

^e^7 Gram-negative organisms were resistant.

^f^1 Gram-negative organism was resistant.

**Table 3:** Antibiotic coverage against the most commonly isolated bacteria.

Pathogen	Antibiotic coverage^#^
*Staphylococcus *species	Cotrimoxazole (28/28), neomycin (17/17)^a^, tobramycin (17/17), doxycycline (22/22), and erythromycin (26/26)

*Pseudomonas aeruginosa*	Gentamicin (19/19), ceftazidime (19/19), ciprofloxacin (19/19), tobramycin (10/10), and neomycin (11/11)^b^

*Streptococcus *species	Erythromycin (18/18), ciprofloxacin (13/13)^c^, penicillin (13/13), chloramphenicol (13/13), cefazolin (11/11), and vancomycin (6/6)

*Moraxella *species	Ciprofloxacin (16/16), cefazolin (12/12), chloramphenicol (10/10), neomycin (9/9), and tobramycin (9/9)

*Corynebacterium *species	Doxycycline (12/12), vancomycin (9/9), tobramycin (6/6), ciprofloxacin (6/6), neomycin (5/5), and penicillin (8/10)

^#^Only selected isolates and antibiotics were tested; ^a^intermediate resistance in one; ^b^in five; and ^c^in six.

**Table 4:** Bacterial isolates and their sensitivity to commonly used antibiotics recorded in New Zealand and UK regions/cities.

	Present study, Auckland (2013-2014)	Christchurch, NZ [16] (1991–2001)	Wellington, NZ [17] (2001–2005)	Waikato, NZ [15] (2003–2007)	Oxford, UK [12] (1999–2009)
Positive culture (bacteria)	57.7% (89/154)	58.6% (51/87)	85.3% (29/34)	65.7% (174/265)	53.7% (252/467)
Gram-positive isolates	57.5% (73/127)	71.4% (45/63)	82.5% (33/40)	78.2% (136/174)	54.3% (145/267)
Gram-negative isolates	42.5% (54/127)	28.5% (18/63)	17.5% (7/40)	20.1% (35/174)	45.7% (122/267)
Ciprofloxacin (%)	98.8	92	N/A	99	92.7
Gentamicin (%)	100	N/A	N/A	N/A	93
Tobramycin (%)	100	N/A	N/A	95	N/A
Neomycin (%)	100	N/A	2.5	N/A	N/A
Chloramphenicol (%)	94.7	96.8	100	93.1	68.1
Cefuroxime (%)	33.3	N/A	N/A	95	49.1

N/A = not available.

**Table 5:** Relative proportions of the five most common pathogens in the present study compared to bacterial keratitis isolates from published reports.

Bacterial species	Present study	Christchurch, NZ [16]	Wellington, NZ [17]	Waikato, NZ [15]	Taiwan [22]	Australia [18]	Auckland [10]
Study period	2013-2014	1997–2001	2001–2005	2003–2007	1994–2005	2001–2003	1999–2001
*Staphylococcus* (%)^*∗*^	38.2	30.5	62.5	52.3	11	46.6	54.6
*Pseudomonas *(%)	21.3	3.1	N/A	3.4	46.7	8.3	9.3
*Moraxella *(%)	18	19.3	12.5	8	N/A	N/A	8
*Corynebacterium *(%)	16.9	16	7.5	6.9	3.3	1.7	8
*Streptococcus *(%)	20.2	17.5	2.5	11.5	7.6	6.7	14.7

^*∗*^Including coagulase-negative *Staphylococcus aureus*.

N/A = not available.
